# Carbohydrate content in the diet of red-bellied pacu (*Piaractus brachypomus*) and its effects on growth performance, nutrient deposition, digestive enzymes, lipogenesis, and glycolysis

**DOI:** 10.1007/s00360-026-01715-7

**Published:** 2026-09-15

**Authors:** Rafael Carvalho da Silva, Carlos Eduardo Copatti, Aline da Silva Rocha, Rafael Silva Marchão, David Ramos da Rocha, Anderson Miranda de Souza, Yuanbing Wu, Isidoro Metón, José Fernando Bibiano Melo

**Affiliations:** 1 Postgraduate Program in Animal Science, Federal University of the São Francisco Valley (UNIVASF), Petrolina, 56300-000 PE Brazil; 2 Postgraduate Program in Zootechny, Federal University of Bahia (UFBA), Salvador, 40170–115 BA Brazil; 3 Secció de Bioquímica i Biologia Molecular, Departament de Bioquímica i Fisiologia, Facultat de Farmàcia i Ciències de l’Alimentació, Universitat de Barcelona (UB), Joan XXIII 27-31, Barcelona, 08028 Spain

**Keywords:** Carbohydrate utilization, Intestinal enzymes, Feed conversion ratio, Gluconeogenesis, Liver enzymes

## Abstract

This study evaluated the effects of varying dietary carbohydrate (CBH) levels on growth performance, body composition, hematological parameters, metabolism, and intestinal and hepatic enzyme activity in juvenile red-bellied pacu (*Piaractus brachypomus*). Fish (initial weight 70.25 g) were fed three times daily to apparent satiety for 45 days with diets containing 371.80–378.20 g kg^–1^ crude protein and 17.00-17.42 MJ kg^–1^ gross energy, and supplemented with 230, 260, 290, 320, or 350 g CBH kg^–1^. Fish were distributed among 15 tanks (practical volume of 800 L; stocking density of 0.878 g L^–1^), with treatments replicated 3 times. Optimal growth performance, feed conversion ratio, and whole-body nutrient deposition were observed in fish fed diets containing 260–290 g CBH kg^–1^. Fish fed the highest CBH level (350 g kg^–1^) exhibited reduced erythrocyte counts but increased hemoglobin concentration. Increasing dietary CBH levels resulted in higher hepatosomatic index, plasma triglyceride concentration, and liver and muscle glycogen stores, while reducing the viscerosomatic index and hepatic aspartate aminotransferase activity, with no effect on glycemia. Intermediate CBH levels (260–320 g kg^–1^) increased intestinal amylase and lipase activities but reduced alkaline protease activity. In the liver, high-CBH diets stimulated the activity of key enzymes involved in glycolysis (6-phosphofructo-1-kinase, PFK1; and pyruvate kinase, PK), the pentose phosphate pathway (6-phosphogluconate dehydrogenase, 6PGDH), and the tricarboxylic acid cycle (isocitrate dehydrogenase, ICDH), while decreasing the activity of rate-limiting enzymes in gluconeogenesis (fructose-1,6-bisphosphatase 1, FBPase1) and amino acid deamination (glutamate dehydrogenase, GDH). In conclusion, dietary CBH levels between 260 and 290 g kg^–1^ are recommended to optimize growth performance and enhance digestive and hematometabolical efficiency in juvenile red-bellied pacu. Exceeding this range may impair performance and welfare, highlighting the importance of precise dietary formulation in aquaculture.

**Supplementary Information:**

The online version contains supplementary material available at https://doi.org/10.1007/s00360-026-01715-7.

## Introduction

Carbohydrates (CBH) are low-cost energy sources commonly included in commercial aquafeeds to reduce production costs while sparing dietary protein whenever possible (Stone 2003; Kamalam et al. 2017; Zhao et al. 2022). In addition, the inclusion of CBH in fish diets can improve pellet physical quality, help preserve body protein and lipid reserves, and provide essential metabolites, such as glycoproteins, glucose, and nucleotides (Kamalam et al. 2017; Sulaiman et al. 2020). When dietary CBH levels are insufficient, however, fish rely more heavily on protein and lipids to meet their metabolic demands (Hemre et al. 2002; Wang et al. 2022). Consequently, there has been growing interest in reducing fishmeal inclusion in aquafeeds by increasing the use of CBH-rich ingredients (Viegas et al. 2016; Macusi et al. 2023). Despite these advantages, many fish species have a limited capacity to efficiently utilize dietary CBH.

The efficiency of CBH utilization in fish depends on several factors, including ingredient type and inclusion level (Sulaiman et al. 2020; Marchão et al. 2025), as well as species-specific feeding habits (Stone 2010; De Souza et al. 2021a). Because dietary CBH are digested relatively slowly, appropriate inclusion levels may promote stable glycemic responses and improved growth, thereby reducing protein and lipid catabolism (Hemre et al. 2002; Zhao et al. 2022). In contrast, excessive dietary CBH intake can lead to prolonged hyperglycemia, metabolic disorders, excessive lipid deposition, and immunosuppression (Kamalam et al. 2017; Walker et al. 2020; Ferrari et al. 2022). In general, omnivorous and herbivorous species tolerate higher CBH levels and utilize CBH-rich diets more efficiently than carnivorous fish (Sandre et al. 2017a; b; De Farias et al. 2021; De Souza et al. 2021a).

Glycolysis and gluconeogenesis are opposite but complementary metabolic pathways involved in CBH utilization and glucose homeostasis (Metón et al. 1999). Adequate dietary CBH levels tend to suppress gluconeogenesis by reducing the activity of key enzymes, such as fructose-1,6-bisphosphatase 1 (FBPase1), as well as amino acid deamination through decreased glutamate dehydrogenase (GDH) activity, thereby limiting glucose production from non-glycosidic precursors (Melo et al. 2016; Carli et al. 2024; Satiro et al. 2024; Thongchaitriwat et al. 2024). This metabolic shift favors energy production via glycolysis (De Farias et al. 2021), the primary cellular pathway for glucose utilization, which generates pyruvate and produces adenosine triphosphate (ATP, a source of energy) and nicotinamide adenine dinucleotide (NADH, a source of reducing power) (Su et al. 2020; Dai et al. 2024). Key hepatic enzymes involved in glycolysis, such as 6-phosphofructo-1-kinase (PFK1) and pyruvate kinase (PK), as well as enzymes of the tricarboxylic acid cycle, including isocitrate dehydrogenase (ICDH), exhibit increased activity in fish fed CBH-rich diets (Rashidpour et al. 2019; Pereira et al. 2020; Su et al. 2020; Carli et al. 2024). The same pattern is observed for the lipogenic enzyme 6-phosphogluconate dehydrogenase (6PGDH) in European seabass (*Dicentrarchus labrax*) and yellow catfish (*Pelteobagrus fulvidraco*) fed CBH-rich diets (Viegas et al. 2016; Yang et al. 2020a). This enzyme is strongly linked to lipid synthesis, as it produces nicotinamide adenine dinucleotide phosphate (NADPH) via the pentose phosphate pathway (Sa et al. 2008).

Digestive enzymes are essential indicators of fish nutritional physiology, as their activities reflect the capacity to digest and utilize dietary nutrients. Amylase and lipase activities may increase in response to elevated dietary CBH levels (Zheng et al. 2023; Marchão et al. 2024, 2025). Complementary physiological assessments, such as hematological analyses, can further elucidate fish responses to CBH-rich diets (Felix e Silva et al. 2020, 2022; De Souza et al. 2021b). Moreover, dysregulation of hepatic lipid metabolism resulting from excessive CBH intake has been linked to the development of fatty liver in fish, which may reduce aspartate aminotransferase (AST) activity and compromise liver function (Yang et al. 2020a; Carli et al. 2024).

The red-bellied pacu (*Piaractus brachypomus*) is an herbivorous species widely farmed in South America and introduced into several Asian countries, including India, Bangladesh, Thailand, and China (Guimarães and Martins 2015). This species has high economic value and is suitable for cultivation in excavated ponds and recirculating aquaculture systems, which encourages technological development and the search for efficient dietary energy sources (Ananias et al. 2025). Previous studies have shown that red-bellied pacu readily accept CBH-rich ingredients (Lochmann et al. 2009) and can utilize dietary CBH more efficiently than lipids, tolerating inclusion levels of up to 380 g kg^–1^ (Vásquez-Torres and Arias-Castellanos 2013). However, these studies primarily focused on growth performance, leaving key gaps in understanding the effects of dietary CBH on hematological parameters, metabolic profiles, and enzymatic activities in key organs, such as the liver and intestine. Together, these analyses provide a comprehensive view of how dietary CBH may influence metabolic and physiological responses to fish.

Therefore, given the importance of enzymatic regulation in determining CBH utilization efficiency, we hypothesized that intermediate dietary CBH levels would optimize growth performance and metabolic efficiency in juvenile red-bellied pacu. Accordingly, this study aimed to evaluate the effects of increasing dietary CBH levels on growth performance, whole-body composition and nutrient deposition, hematological parameters, metabolic indicators, and the activity of hepatic and digestive enzymes in this species.

## Materials and methods

### Animals, acclimatization, and experimental conditions

All procedures involving animals were performed in accordance with ethical standards and were approved by the Ethics Committee on Animal Use of the Federal University of Vale do São Francisco (UNIVASF), Petrolina, PE, Brazil (protocol no. 0002/260722).

Juvenile red-bellied pacus were obtained from a commercial hatchery in Aracaju, SE, Brazil, and transported to the UNIVASF Aquaculture Laboratory in water-filled, oxygenated containers. Upon arrival, fish were acclimated for 15 days in five 1,000-L circular polyethylene tanks (800 L of practical volume). The recirculating aquaculture system was equipped with aeration and mechanical and biological filtration, maintaining a water flow rate of 160 L h^–1^. During the acclimation period, fish were fed a commercial extruded diet (32% crude protein; Guabi^®^, Petrolina, PE, Brazil) three times daily (08:00, 12:00, and 16:00 h) to apparent satiety.

After acclimation, 150 fish (initial weight: 70.25 ± 1.43 g) were randomly distributed into 15 tanks (stocking density: 0.878 g L^–1^), following a completely randomized design with five dietary treatments and three replicates per treatment (*n* = 10 fish per tank). Fish were weighed individually before being distributed to the tanks. The feeding routine during the experimental period was identical to that used during acclimation. Apparent satiety was determined by direct observation and was considered achieved when fish ceased actively searching for feed. Feed intake per experimental unit was measured every three days as the difference in diet weight in the containers.

Tanks were cleaned daily, 30 min after the last feeding. Water quality parameters were monitored daily at 10:00 h. They remained stable throughout the experiment: temperature 26.6 ± 1.02 °C (mercury thermometer), dissolved oxygen 5.60 ± 0.15 mg L^–1^ (HI 9146 oximeter, Hanna^®^), pH 7.05 ± 0.21 (HI 8424 pH meter, Hanna^®^), and un-ionized ammonia 0.014 ± 0.03 mg L^–1^ (Alfakit^®^ kit, Florianópolis, SC, Brazil). The experimental period lasted 45 days, during which fish remained healthy and showed no signs of disease.

### Diet formulation and preparation

Five experimental diets (Table 1) were formulated with increasing levels of CBH (230, 260, 290, 320, and 350 g kg^–1^), while maintaining crude protein and gross energy at 371.80–378.20 g kg^–1^ and 17.00-17.42 MJ kg^–1^, respectively. All ingredients were ground to pass through a 1.0-mm sieve, weighed, and thoroughly mixed. The mixtures were moistened with 30% water and extruded (Inbramaq^®^, AM25, Ribeirão Preto, SP, Brazil) using a 5.0-mm die. Pellets were dried at 55 °C for 24 h, packaged in polyethylene bags, and stored at − 20 °C until use.

**Table 1:** Formulation and chemical composition of experimental diets with graded carbohydrate levels for red–bellied pacu (*Piaractus brachypomus*)

Ingredients	Carbohydrates (g kg^–1^)				
	230	260	290	320	350
Fish meal (54%)	498.41	385.36	272.30	159.25	46.20
Toasted soybean meal (45%)	30.00	175.40	320.80	466.20	611.60
Corn bran	57.65	50.74	43.82	36.91	30.00
Wheat bran	150.00	120.00	90.00	60.00	30.00
Corn starch	108.40	116.08	123.76	131.43	139.11
Soy oil	50.00	50.00	50.00	50.00	50.00
Lysine–HCL 78.40%	3.23	3.08	2.94	2.80	2.66
DL–Methionine 99%	0.00	0.47	0.94	1.41	1.89
Dicalcium phosphate	0.00	7.14	14.28	21.42	28.56
Inert (Caulim 2810^®^)	94.61	84.03	73.45	62.87	52.29
Salt	2.00	2.00	2.00	2.00	2.00
Premix^1^	5.00	5.00	5.00	5.00	5.00
Vitamin C^2^	0.50	0.50	0.50	0.50	0.50
Antifungal	0.10	0.10	0.10	0.10	0.10
Antioxidant (BHT)^3^	0.10	0.10	0.10	0.10	0.10
Analyzed proximate composition (g kg^–1^)					
Carbohydrates^4^	235.50	266.00	292.80	321.80	351.50
Dry matter	956.80	958.86	960.02	962.10	964.00
Crude protein	371.80	374.80	373.40	374.10	378.20
Ether extract	99.20	94.40	87.50	81.30	80.20
Crude fiber	74.90	72.80	67.10	64.20	62.80
Ash	218.60	192.00	179.20	158.60	127.70
Crude energy (MJ kg^–1^)	17.10	17.00	17.11	17.21	17.42
Calculated amino acids (g kg**–1**)^5^					
Lysine	21.38	21.31	21.24	21.17	21.10
Methionine	7.42	7.15	6.88	6.62	6.35
Threonine	13.05	12.84	12.63	12.42	12.21
Tryptophan	2.90	3.21	3.53	3.85	4.16
Valine	16.04	15.68	15.32	14.96	14.60
Isoleucine	12.76	13.13	13.50	13.87	14.24
Histidine	6.56	6.95	7.33	7.72	8.10
Leucine	22.48	22.82	23.16	23.50	23.84
Arginine	19.60	20.32	21.04	21.76	22.48
Phenylalanine	13.04	13.72	14.40	15.08	15.76

^1^ Vitamin and mineral premix (Vitaly^®^, Feira de Santana, BA, Brazil) (guaranteed levels per kg of premix): Vit. A, 1,200,000 UI; Vit. D3, 200,000 UI; Vit. E, 12,000 mg; Vit. K3, 2,400 mg; Vit. B1, 4,800 mg; Vit. B2, 4,800 mg; Vit. B6, 4,000 mg; Vit. B12, 4,800 mg; folic acid, 1,200 mg; Ca pantothenate, 12,000 mg; Vit. C, 48,000 mg; biotin, 48 mg; choline chloride, 65,000 mg; niacin, 24,000 mg; Fe, 10,000 mg; Cu, 6,000 mg; Mn, 4,000 mg; Zn, 6,000 mg; I, 20 mg; Co, 2 mg; Se, 20 mg

^2^ Protected Vit. C (Vitaly^®^): Ca salt, active ingredient 42% ascorbic acid 2 2-monophosphate

^3^ Butylated hydroxytoluene

^4^ Calculated values of carbohydrates from proximate composition analyses

^5^ The total amino acid content was calculated after analyzing the amino acids of the ingredients by HPLC at CBO (Valinhos, SP, Brazil)

The CBH was calculated from the ingredient composition of the experimental diets, according to NRC (2011) and to Rostagno and Albino (2024). For this study, the following CBH values were adopted: soybean meal, 30.71%; corn bran, 72.95%; wheat bran, 55.06%; and corn starch, 86.00%. Total dietary CBH was calculated as the weighted sum of the inclusion level of each ingredient, considering its proportional contribution to the formulation and its respective content of non-structural CBH, such as starch, sugars, and pectins. Toasted soybean meal (45% crude protein) was used, as thermal processing inactivates major antinutritional factors in soybeans (e.g., protease inhibitors, urease, and lectins). Protein and energy levels were determined based on other previously published studies (Guimarães and Martins 2015; Favero et al. 2021). The CBH was calculated according to Zhang et al. (2020): CBH = 100 − (crude protein + crude lipid + crude fiber + ash).

Proximate composition analyses were performed according to Association of Official Agricultural Chemists (AOAC 2016): dry matter was determined by oven-drying at 100–105 °C to constant weight; crude protein by nitrogen determination using the Kjeldahl method (Quimis^®^, Diadema, SP, Brazil) and multiplication by 6.25; total lipids by ether extraction under high pressure and temperature (Luca 202, Lucadema^®^, São José do Rio Preto, SP, Brazil); crude fiber by sequential acid and alkaline digestion; ash by combustion in a muffle furnace (TE–1100–1P, Tecnal^®^, Piracicaba, SP, Brazil) at 550 °C for 4 h; and gross energy by adiabatic bomb calorimetry (Parr 1261^®^, Moline, IL, USA).

The total amino acid content of the diets was calculated from amino acid analyses of the ingredients by high-performance liquid chromatography (HPLC) at the CBO Laboratory (Valinhos, SP, Brazil). The amino acid profile of the experimental diets was adequate to meet the essential amino acid requirements of pacu (*Piaractus mesopotamicus*) (Khan et al. 2020), a round fish also belonging to the genus *Piaractus*.

### Productive performance

The fish were fed for 45 days, then feeding was stopped. On day 46 (24 h after the last feeding), they were anesthetized with benzocaine (50 mg L^–1^) and individually weighed. Performance metrics were calculated as follows:

$$ {\text{Final weight (FW, g) = fish weight at the end of the experimental period;}} $$

$$ {\text{Weight gain (WG, g) = final weight (g) }} $$

$$ \:{\mathrm{Relative}}\:{\mathrm{weight}}\:{\mathrm{gain}}\:({\mathrm{RWG}},\:\% )\:\:{\text{ = }}\:\:{\mathrm{100}}\: \times \:\frac{{{\mathrm{final}}\:{\mathrm{weight}}\:({\mathrm{g}})\:{\mathrm{0}}\:{\mathrm{initial}}\:{\mathrm{weight}}\:({\mathrm{g}})}}{{{\mathrm{initial}}\:{\mathrm{weight}}\:\left( {\mathrm{g}} \right)}} $$

$$ \:{\mathrm{Specific}}\:{\mathrm{growth}}\:{\mathrm{rate}}\:({\mathrm{SGR}},\:\% \:{\text{day - 1}})\:{\text{ = }}\:{\mathrm{100}}\: \times \:\:\frac{{{\mathrm{ln}}\:{\mathrm{final}}\:{\mathrm{weight}}\:({\mathrm{g}})\:{\mathrm{0}}\:{\mathrm{ln}}\:{\mathrm{initial}}\:{\mathrm{weight}}\:({\mathrm{g}})}}{{{\mathrm{time}}\:\left( {{\mathrm{days}}} \right)}} $$

$$\:\mathrm{Feed\:intake\:(FI,\:g\:fish}\mathrm{\--1}\mathrm{)\:=\:}\frac{\mathrm{t}\mathrm{o}\mathrm{t}\mathrm{a}\mathrm{l}\:\mathrm{c}\mathrm{o}\mathrm{n}\mathrm{s}\mathrm{u}\mathrm{m}\mathrm{e}\mathrm{d}\:\mathrm{f}\mathrm{e}\mathrm{e}\mathrm{d}\:\left(\mathrm{g}\right)}{\mathrm{n}\mathrm{u}\mathrm{m}\mathrm{b}\mathrm{e}\mathrm{r}\:\mathrm{o}\mathrm{f}\:\mathrm{f}\mathrm{i}\mathrm{s}\mathrm{h}\:\mathrm{p}\mathrm{e}\mathrm{r}\:\mathrm{t}\mathrm{a}\mathrm{n}\mathrm{k}}$$

$$\:\mathrm{Feed\:conversion\:ratio\:}\mathrm{(FCR)\:=\:}\frac{\mathrm{f}\mathrm{e}\mathrm{e}\mathrm{d}\:\mathrm{i}\mathrm{n}\mathrm{t}\mathrm{a}\mathrm{k}\mathrm{e}\:\left(\mathrm{g}\right)}{\mathrm{w}\mathrm{e}\mathrm{i}\mathrm{g}\mathrm{h}\mathrm{t}\:\mathrm{g}\mathrm{a}\mathrm{i}\mathrm{n}\:\left(\mathrm{g}\right)}$$

$$ \:{\mathrm{Carcassyield}}\:({\mathrm{CY}},\:\% )\:\:{\text{ = }}\:\:{\mathrm{100}}\: \times \:\frac{{{\mathrm{carcass}}\:{\mathrm{weight}}\:({\mathrm{g}})}}{{{\mathrm{total}}\:{\mathrm{body}}\:{\mathrm{weight}}\:\left( {\mathrm{g}} \right)}} $$

$$ \:{\mathrm{Hepatosomatic}}\:{\mathrm{index}}\:({\mathrm{HSI}},\:\% )\:\:{\text{ = }}\:\:{\mathrm{100}}\: \times \:\frac{{{\mathrm{liver}}\:{\mathrm{weight}}\:({\mathrm{g}})}}{{{\mathrm{total}}\:{\mathrm{body}}\:{\mathrm{weight}}\:\left( {\mathrm{g}} \right)}} $$

$$ \:{\mathrm{Viscerosomatic}}\:{\mathrm{index}}\:({\mathrm{VSI}},\:\% )\:\:{\text{ = }}\:\:{\mathrm{100}}\: \times \:\frac{{{\mathrm{viscera}}\:{\mathrm{weight}}\:({\mathrm{g}})}}{{{\mathrm{total}}\:{\mathrm{body}}\:{\mathrm{weight}}\:\left( {\mathrm{g}} \right)}} $$

The CY, HSI, and VSI were determined in two fish per replicate (*n* = 6 per treatment), euthanized with benzocaine (250 mg L^–1^) and spinal cord transection.

### Tissue collection

On day 46, two fish were randomly selected from each tank (*n* = 6 per treatment) for tissue collection. Fish were anesthetized (benzocaine, 50 mg L^–1^), and 2.0 mL of blood was drawn from the caudal vein using heparinized syringes (5,000 IU). Samples were split for hematological and plasma analyses. Plasma was separated by centrifugation at 3,000 × g for 10 min at 4 °C and stored at − 20 °C.

After blood collection, fish were euthanized (250 mg benzocaine L^–1^ and spinal cord severance). White muscle, liver, viscera, carcass, and intestine were excised, weighed, and stored at − 80 °C. Livers were divided (~ 2.60 g each): one portion (100 mg) was used fresh for protein, AST, and glycogen assays, while the other was freeze-dried (LS 3000) for enzymatic analysis. Muscle (100 mg) and intestine (100 mg) were used for glycogen and enzymatic analysis, respectively.

### Whole-body composition and nutrient deposition

At the start, 12 non-experimental fish were euthanized (250 mg benzocaine L^–1^, spinal cord section) for baseline composition. Similarly, at the end, two fish were sampled per tank (*n* = 6 per treatment). All sampled fish were fasted for 24 h before processing.

Samples were thawed, pre-dried at 65 °C for 72 h, ground using an industrial mill (Marconi MA923^®^, São Paulo, SP, Brazil), and stored in sealed containers. Analyses followed the AOAC (2016) guidelines as outlined in Section “Diet formulation and preparation”. Nutrient deposition was calculated as follows:

$$\:\mathrm{Body\:protein\:deposition\:(BPD,\:g)\:=\:final\:body\:protein\:\--\:initial\:body\:protein\:}$$

$$\:\mathrm{Body\:fat\:deposition\:(BFD,\:g)\:=\:final\:body\:fat\:\--\:initial\:body\:fat}$$

$$\:\mathrm{Body\:ash\:deposition\:(BAD,\:g)\:=\:final\:body\:ash\:matter\:\--\:initial\:body\:ash\:matter}$$

### Hematological analyses

Blood was analyzed immediately. Erythrocyte (Ery) counts were performed in a Neubauer chamber at 400× magnification (Natt and Herrick 1952). Hematocrit (Ht) was measured using a microhematocrit method (Goldenfarb et al. 1971) with heparinized capillaries, centrifuged at 12,000 × g for 5 min at 4 °C (SH–120 microcentrifuge, Logen Scientific). Hemoglobin (Hb) was determined by the cyanmethemoglobin method (Drabkin 1948). Hematimetric indices were calculated as:

$$ {\mathrm{Mean}}\:{\mathrm{corpuscular}}\:{\mathrm{volume}}\:({\mathrm{MCV}},\:{\mathrm{fL}})\:{\text{ }} = {\text{ }}\:\frac{{{\mathrm{Ht}}\:{\mathrm{x}}\:10}}{{{\mathrm{Ery}}\left( { \times 10^{6} \mu {\mathrm{L}}} \right)}}$$$$$$$$ $$

$$\:\mathrm{Mean\:corpuscular\:hemoglobin\:concentration\:(MCHC,\:g\:dL}\mathrm{\--1}\mathrm{)\:=\:}\frac{\mathrm{Hb\:x\:100}}{\mathrm{H}\mathrm{t}}$$

$$ {\mathrm{Mean}}\:{\mathrm{corpuscular}}\:{\mathrm{hemoglobin}}\:({\mathrm{MCH,}}\:{\mathrm{pg}})\:{\text{ }} = {\text{ }}\:\frac{{{\mathrm{Hb}}\:{\mathrm{x}}\:10}}{{{\mathrm{Ery}}\:\left( { \times 10^{6} \:\mu {\mathrm{L}}} \right)}} $$

### Plasmatic, hepatic, and muscle analysis

Plasma glucose, cholesterol, triglycerides, total protein, and albumin were measured using commercial colorimetric kits (Labtest^®^, Vista Alegre, MG, Brazil). Absorbance was measured at 340, 500, 505, 545, and 600 nm using a spectrophotometer (Biospectro SP 2000 UV). Liver and muscle glycogen were determined according to the method described by Bidinotto et al. (1997). Hepatic protein and AST were measured with Labtest^®^ kits at 340 nm and 545 nm. Tissues were homogenized in 10 mM phosphate/20 mM Tris buffer (pH 7.0, 4 °C) using a mechanical homogenizer (Marconi MA039^®^).

Enzymatic activities of hepatic PFK1, PK, FBPase1, 6PGDH, GDH, and ICDH were measured spectrophotometrically at 30 °C and 340 nm using a Varioskan Lux multimode microplate reader (Thermo Fisher Scientific^®^, Waltham, MA, USA). PFK1, PK, FBPase1, and 6PGDH were assayed according to Metón et al. (1999), while GDH and ICDH were assayed as described by Rashidpour et al. (2019). Before performing these analyses, the Bradford method (1976) was used to determine the total soluble protein concentration, with bovine serum albumin serving as a standard. The crude extracts for the determination of enzyme activities were obtained by homogenization of previously lyophilized liver tissue (1:10, w/v) in 50 mM Tris-HCl (pH 7.5), ethylenediamine tetra acetic acid 4 mM, NaF 50 mM, phenylmethylsulphonyl fluoride 0.5 mM, dithiothreitol 1 mM and sacarose 250 mM, by 30 s at 4 °C, using a homogenizer (PTA-7 Polytron, Kinematica GmbH^®^, Littau-Luzern, Switzerland). Subsequently, the samples were centrifuged at 10,000 × g for 30 min at 4 °C, and the supernatant was collected for enzyme activity assays. All enzyme activities are expressed as U mg^–1^ protein.

### Intestinal enzyme activity

The anterior small intestine was taken 4 cm distal to the stomach. Then, the tissue was homogenized in 10 mM phosphate/20 mM Tris buffer (pH 7.0 at 4 °C) using a mechanical homogenizer (Marconi MA039^®^). Supernatants were used for enzyme determinations. Amylase and lipase activities were quantified using commercial kits (BioClin^®^, Belo Horizonte, MG, Brazil) with absorbance readings at 405 nm and 410 nm, respectively.

Alkaline protease activity was assayed following Sarath et al. (1989), using 0.1 M Tris/HCl buffer (pH 8.5) and 1% casein. Reactions were incubated at 30–35 °C, stopped with 15% trichloroacetic acid, and centrifuged at 1,800 × g for 10 min. Tyrosine served as a standard, with absorbance measured at 420 nm.

### Statistical analysis

Statistical analyses were performed using InfoStat^®^ (2010). Data normality and homogeneity were checked using the Shapiro–Wilk and Levene tests (*P* < 0.05). One-way analysis of variance (ANOVA) was used, followed by Tukey’s post-hoc test (*P* < 0.05). For significant effects (*P* < 0.05), orthogonal polynomial contrasts were used to examine linear and quadratic trends in dietary CBH levels, and model selection was based on the highest R² value. Estimating the CBH requirements also involved the use of derived quadratic equations for growth performance and whole-body deposition.

## Results

### Productive performance, whole-body composition, and nutrient deposition

The fish had no mortality. The regression analysis revealed that dietary CBH increase was associated with a linear increase in HSI (*P* < 0.05). The quadratic response was found to augment final weight, weight gain, RWG, SGR, and VSI, and to reduce FCR and CY when fish were fed 260 and 290 g CBH kg^–1^ (*P* < 0.05) (Table 2).

**Table 2:** Zootechnical parameters of red–bellied pacu (*Piaractus brachypomus*) fed diets with different levels of carbohydrate inclusion for 45 days

Variables	Carbohydrates (g kg^–1^)						ANOVA	Regression (*p*)		
	230	260	290	320	350	SEM	*p*-value	Lin	Quadr	*R^2^
IW	70.17	70.28	69.24	70.78	70.77	0.22	0.159	0.182	0.248	–
FW^1^	272.50^a^	275.89^a^	276.51^a^	262.30^a^	241.31^b^	3.91	0.001	< 0.001	0.003	0.81
WG^2^	202.49^a^	205.60^a^	207.27^a^	191.52^a^	170.54^b^	3.98	0.001	< 0.001	0.002	0.83
RWG^3^	289.22^ab^	292.52^ab^	299.33^a^	270.59^b^	241.00^c^	6.01	< 0.001	< 0.001	0.001	0.85
SGR^4^	2.34^ab^	2.36^ab^	2.39^a^	2.26^b^	2.12^c^	0.03	< 0.001	< 0.001	0.001	0.85
FI	225.31	229.52	230.73	232.30	223.30	1.41	0.337	0.065	0.577	–
FCR^5^	1.11^c^	1.12^bc^	1.11^bc^	1.21^b^	1.39^a^	0.03	< 0.001	< 0.001	< 0.001	0.90
CY^6^	86.39^ab^	86.09^bc^	85.85^c^	86.16^bc^	86.74^a^	0.07	0.005	0.012	< 0.001	0.68
VSI^7^	13.54^b^	13.99^ab^	14.44^a^	13.93^b^	12.76^c^	0.12	< 0.001	< 0.001	< 0.001	0.80
HSI^8^	1.55^c^	1.70^bc^	1.88^ab^	2.05^a^	2.06^a^	0.05	< 0.001	< 0.001	0.227	0.59

IW (initial weight), FW (final weight), and WG (weight gain) are expressed as g. RWG (relative weight gain), CY (carcass yield), VSI (viscerosomatic index), and HSI (hepatosomatic index) are expressed as %. SGR (specific growth rate) is expressed as %/day^–1^. FI (feed intake) is expressed as g fish^–1^. FCR = feed conversion ratio. SEM = standard error of the mean. *N* = 3 tanks per treatment. Different letters indicate the statistical difference between treatments (Tukey’s test, *P* < 0.05). * Regression equations (the model with the highest R² value was chosen): Y^1^ = – 0.00505 × ^2^ + 2.6737x – 76.183; Y^2^ = – 0.00521 × ^2^ + 2.7602x – 157.680; Y^3^ = – 0.00804 × ^2^ + 4.270x – 268.933; Y^4^ = – 0.00004 × ^2^ + 0.0201x – 0.283; Y^5^ = 0.00003 × ^2^ – 0.0173x + 3.312; Y^6^ = 0.00018x² – 0.1047x + 100.717; Y^7^ = – 0.00033x² + 0.1883x – 12.186; Y^8^ = 0.0044x + 0.560

According to Tukey’s test, the group fed with 350 g CBH kg^–1^ had significantly lower final weight, weight gain, RWG, SGR, and VSI values than the other groups (*P* < 0.05). In addition, dietary CBH inclusion at 320 g kg^–1^ promoted significantly lower values for RWG, SGR, and VSI (also for 230 g kg^–1^) than at 290 g kg^–1^ (*P* < 0.05). Feeding with a 350 g CBH kg^–1^ also resulted in significantly higher values than other treatments for FCR, CY, and HSI (also for 320 g kg^–1^) (*P* < 0.05). No significant differences were found in initial weight and feed intake between treatments (*P* > 0.05) (Table 2).

The regression analysis revealed a linear decrease in ash and BAD levels as CBH level in the diet increased (*P* < 0.05). The variables total lipids, BPD, and BFD exhibited a quadratic response, with the highest values observed in treatments 260 and 290 g CBH kg^–1^ (*P* < 0.05).

In addition, according to Tukey’s test, 290 g CBH kg^–1^ treatment led to significantly higher total lipid values than any other treatment (*P* < 0.05). Fish fed 260–290 g CBH kg^–1^ generally promoted higher BPD and BFD values than those fed 320–350 g CBH kg^–1^ (*P* < 0.05). BAD values were significantly higher in fish fed diets with CBH addition at 230–260 g kg^–1^ than those with CBH addition at 320–350 g kg^–1^ (*P* < 0.05). No significant differences were found between treatments for moisture and crude protein (*P* > 0.05) (Table 3).

**Table 3:** Whole–body composition and deposition of red–bellied pacu (*Piaractus brachypomus*) fed diets with different levels of carbohydrate inclusion for 45 days

Variables		Carbohydrates (g kg^–1^)						ANOVA	Regression (*p*)		
	IC	230	260	290	320	350	SEM	*p*-value	Lin	Quadr	*R^2^
Moisture	67.81	68.90	68.30	68.31	71.29	69.43	0.54	0.419	0.307	0.920	–
CP	19.12	17.16	17.90	17.58	17.77	17.92	0.19	0.769	0.369	0.704	–
TL^1^	8.08	8.90^b^	9.14^b^	10.07^a^	8.31^b^	8.30^b^	0.19	< 0.001	0.005	0.001	0.47
Ash^2^	3.99	3.67^ab^	3.81^a^	3.36^ab^	3.22^b^	3.34^b^	0.07	0.012	0.003	0.503	0.45
BPD^3^	–	33.3^ab^	35.3^a^	35.35^a^	32.68^ab^	29.63^b^	0.65	0.004	0.004	0.003	0.74
BFD^4^	–	18.5^bc^	19.5^ab^	22.27^a^	16.10^cd^	14.31^d^	0.28	< 0.001	< 0.001	< 0.001	0.69
BAD^5^	–	7.21^a^	7.71^a^	6.52^ab^	5.64^b^	5.23^b^	0.28	0.001	< 0.001	0.238	0.68

Moisture, CP (crude protein), TL (total lipids), and ash are expressed as %. BPD (body protein deposition), BFD (body fat deposition), and BAD (body ash deposition) are described as g. IC = initial composition. SEM = standard error of the mean. *N* = 3 tanks per treatment. Different letters indicate the statistical difference between treatments (Tukey’s test, *P* < 0.05). * Regression equations (the model with the highest R² value was chosen): Y^1^ = – 0.00025x² + 0.1404x – 9.979; Y^2^ = – 0.000042x + 0.4690; Y^3^ = – 0.00101 × ^2^ + 0.5527x – 40.099; Y^4^ = – 0.00114 × ^2^ + 0.6237x – 64.422; Y^5^ = – 0.0201x + 12.289

**Fig. 1:**
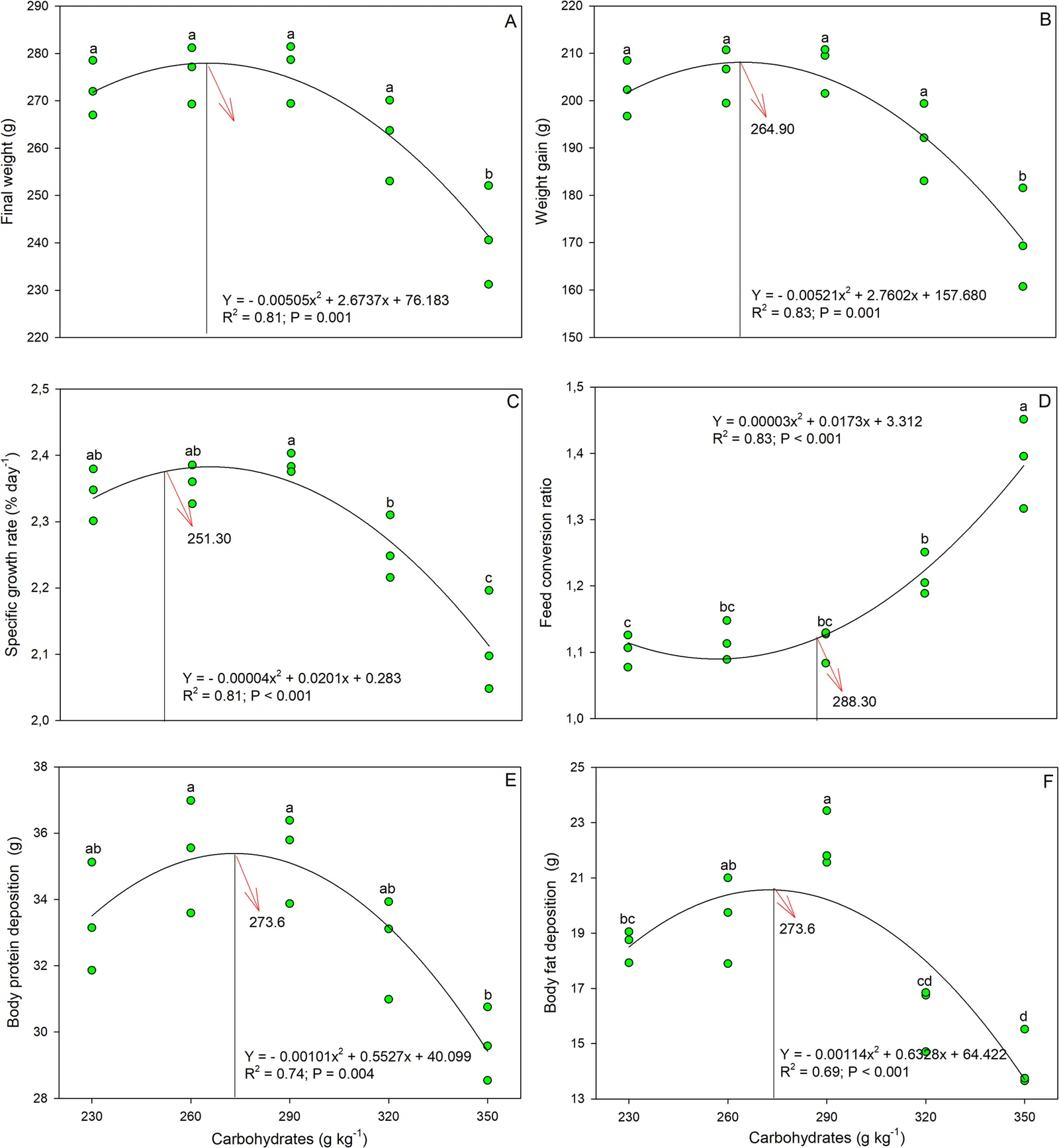
Graphical representation (estimations with derived quadratic regression models) of final weight (**A**), weight gain (**B**), specific growth rate (**C**), feed conversion ratio (**D**), body protein deposition (**E**), and body fat deposition (**F**) of red-bellied pacu (*Piaractus brachypomus*) fed diets with different levels of carbohydrate inclusion for 45 days. *N* = 3 tanks per treatment. Different letters indicate the statistical difference between treatments (Tukey’s test, *P* < 0.05)

The estimated requirement (calculated by the second-order regression) of dietary CBH for final weight, weight gain, SGR, and FCR was 264.7, 264.9, 251.3, and 288.3 g kg^–1^, respectively (Fig. 1a-d). The estimated requirement of dietary CBH for BPD and BFD was 273.6 g kg^–1^ (Fig. 1e-f).

### Hematological and biochemical variables

The hematological responses were analyzed using quadratic regression, where fish receiving 350 g CBH kg^–1^ had the highest hemoglobin and MCV values, as well as the lowest erythrocyte and MCHC values (*P* < 0.05).

In addition, according to Tukey’s test, the fish that received 260 g CBH kg^–1^ for erythrocyte count had values significantly lower than those that received 320 and 350 g CBH kg^–1^ (*P* < 0.05). Fish fed 350 g CBH kg^–1^ had a significantly higher hemoglobin concentration than those fed 320 g CBH kg^–1^ (*P* < 0.05). The treatment of 290 g CBH kg^–1^ showed values significantly higher for MCV and MCHC than the treatments of 260 and 350 (only for MCHC) g CBH kg^–1^ (*P* < 0.05). No significant differences were found for hematocrit and MCH (*P* > 0.05) (Table 4).

**Table 4:** Hematological parameters of red–bellied pacu (*Piaractus brachypomus*) fed diets with different levels of carbohydrate inclusion for 45 days

Variables	Carbohydrates (g kg^–1^)						ANOVA	Regression (*p*)		
	230	260	290	320	350	SEM	*p*–value	Lin	Quadr	*R^2^
Ht	32.67	32.33	32.50	31.67	31.83	0.26	0.740	0.240	0.999	–
Ery^1^	3.04^ab^	3.58^a^	2.94^abc^	2.88^bc^	2.39^c^	0.10	< 0.001	< 0.001	0.019	0.42
Hb^2^	9.38^ab^	9.24^ab^	9.11^ab^	8.91^b^	9.66^a^	0.08	0.012	0.601	0.003	0.27
MCV^3^	107.87^ab^	92.03^b^	135.61^a^	111.08^ab^	113.84^ab^	3.90	0.004	0.003	0.042	0.42
MCH	31.32	31.38	31.42	30.22	30.10	1.04	0.301	0.155	0.133	–
MCHC^4^	29.04^ab^	26.39^bc^	29.32^a^	27.69^abc^	25.76^c^	0.39	0.004	0.406	< 0.001	0.42

Ht (hematocrit) is expressed as %. Ery (erythrocytes) is expressed as ×10^6^ µL. Hb (hemoglobin concentration) and MCHC (mean corpuscular hemoglobin concentration) are expressed as g dL^–1^. MCV (mean corpuscular volume) is expressed as fL. MCH (mean corpuscular hemoglobin) is expressed as pg. SEM = standard error of the mean. *N* = 3 tanks per treatment. Different letters indicate the statistical difference between treatments (Tukey’s test, *P* < 0.05). * Regression equations (the model with the highest R² value was chosen): Y^1^ = – 0.00012x² + 0.0602x – 4.591; Y^2^ = 0.00021x² – 0.1256x + 27.151; Y^3^ = 0.00446x² – 2.3363x + 406.806; Y^4^ = 0.00066x² – 0.3822x + 82.226

The regression analysis showed that as dietary CBH levels increased, plasma triglycerides and hepatic glycogen rose linearly (*P* < 0.05). The quadratic response showed decreased liver AST activity and increased muscle glycogen levels when fish were fed with 290–320 g CBH kg^–1^ (*P* < 0.05) (Table 5).

**Table 5:** Biochemical parameters of red–bellied pacu (*Piaractus brachypomus*) fed diets with different levels of carbohydrate inclusion for 45 days

Variables	Carbohydrates (g kg^–1^)						ANOVA	Regression (*p*)		
	230	260	290	320	350	SEM	*p*-value	Lin	Quadr	*R^2^
Plasma										
Glucose	142.35	139.29	161.01	139.34	146.64	3.30	0.196	0.703	0.400	–
Total protein	2.65	2.56	2.64	2.47	2.66	0.03	0.327	0.779	0.271	–
Albumin	1.44	1.44	1.57	1.23	1.25	0.05	0.219	0.116	0.337	–
Triglycerides^1^	134.56^c^	129.80^c^	156.35^bc^	187.54^ab^	197.38^a^	6.10	< 0.001	< 0.01	0.279	0.62
Cholesterol	142.35	139.29	161.01	139.34	146.64	3.30	0.362	0.703	0.400	–
Liver										
Total protein	2.13	2.47	2.46	2.9	2.46	0.11	0.337	0.233	0.209	–
Glycogen^2^	3.35^d^	3.52^cd^	3.73^bc^	3.88^b^	4.36^a^	0.07	< 0.001	< 0.01	0.165	0.75
AST^3^	80.09^a^	73.26^b^	63.45^c^	63.91^c^	66.73^c^	1.30	< 0.001	< 0.01	< 0.001	0.78
Muscle										
Glycogen^4^	0.92^d^	1.01^cd^	1.16^ab^	1.26^a^	1.07^bc^	0.03	< 0.001	0.001	< 0.001	0.57

Glucose, Triglycerides, and cholesterol are expressed as mg dL^–1^. Total protein and albumin are expressed as g dL^–1^. AST (aspartate aminotransferase) is expressed as U mg protein^–1^. Glycogen is expressed as nmol glucose g tissue^–1^. SEM = standard error of the mean. *N* = 3 tanks per treatment. Different letters indicate the statistical difference between treatments (Tukey’s test, *P* < 0.05). * Regression equations (the model with the highest R² value was chosen): Y^1^ = 0.611x – 16.150; Y^2^ = 0.0079x + 1.484; Y^3^ = 0.00235 × ^2^ – 1.4812x + 297.470; Y^4^ = – 0.00005x² + 0.0298x – 3.423

Moreover, Tukey’s test analysis showed that dietary addition of CBH at 350 g kg^–1^ significantly increased plasma triglyceride levels (except at 320 g kg^–1^) and hepatic glycogen (*P* < 0.05). The quadratic regression and Tukey test showed the highest hepatic AST activity in the 230 g CBH kg^–1^ treatment, followed by the 260 g CBH kg–1 treatment (*P* < 0.05). Fish fed a 320 g CBH kg^–1^ had significantly higher muscle glycogen levels than those fed 230, 260, and 350 g CBH kg^–1^ (*P* < 0.05). In addition, this parameter was significantly higher in fish fed a 290 g CBH kg^–1^ treatment than in those fed 230–260 g CBH kg^–1^ treatments (*P* < 0.05). No significant differences were found for plasma glucose, total protein, albumin, total cholesterol, and hepatic total protein levels (*P* > 0.05) (Table 5).

### Hepatic and intestinal enzyme activity

**Fig. 2:**
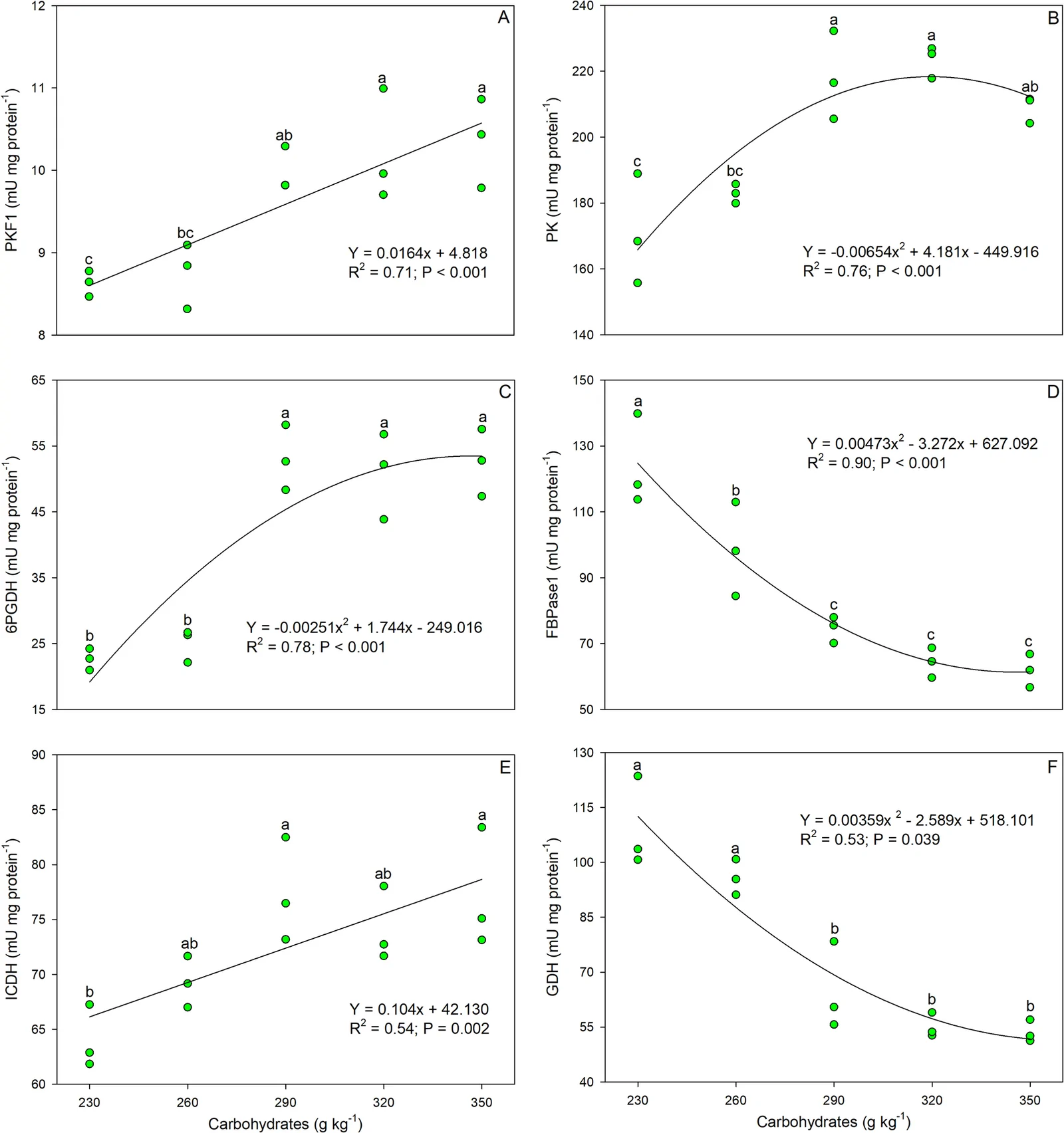
Activity of hepatic enzymes 6-phosphofructo–1-kinase (PFK1) (**A**), pyruvate kinase (PK) (**B**), 6-phosphogluconate dehydrogenase (6PGDH) (**C**), fructose-1,6‐bisphosphatase 1 (FBPase1) (**D**), isocitrate dehydrogenase (ICDH) (**E**), and glutamate dehydrogenase (GDH) (**F**) of red-bellied pacu (*Piaractus brachypomus*) fed diets with different levels of carbohydrate inclusion for 45 days. *N* = 3 tanks per treatment. Different letters indicate the statistical difference between treatments (Tukey’s test, *P* < 0.05)

**Fig. 3:**
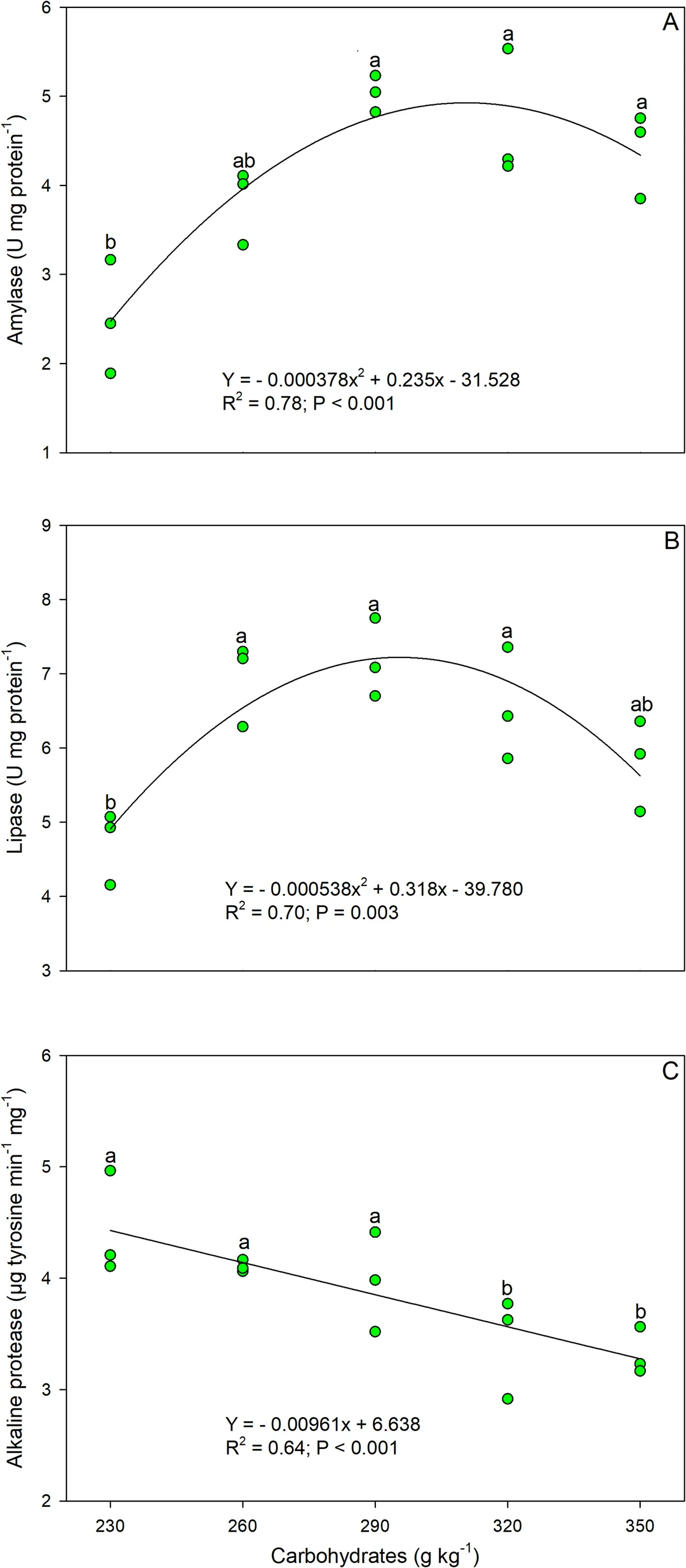
Activity of intestinal enzymes amylase (**A**), lipase (**B**), and alkaline protease (**C**) of red-bellied pacu (*Piaractus brachypomus*) fed diets with different levels of carbohydrate inclusion for 45 days. *N* = 3tanks per treatment. Different letters indicate the statistical difference between treatments (Tukey’s test, *P* < 0.05)

The enzymatic activity responses were analyzed using linear and quadratic regression. As the level of CBH in the diet increased, there was a steady rise in the liver activity of PFK1 and ICDH (*P* < 0.05). The quadratic response for liver enzyme activities showed that fish fed 350 g CBH kg^–1^ presented the highest PK and 6PDGH levels, and the lowest FBPase1 and GDH values (*P* < 0.05) (Fig. 2). An increase in dietary CBH levels was associated with a linear decrease in intestinal alkaline protease activity (*P* < 0.05) (Fig. 3).

According to Tukey’s test, in general, the treatments 230 and 260 g CBH kg^–1^ showed significantly higher hepatic enzyme activities for PFK1, PK, 6PGDH, and ICDH, and significantly lower levels for FBPase1 and GDH than other treatments (*P* < 0.05) (Fig. 2). The treatments 320 and 350 g CBH kg^–1^ significantly lowered intestinal alkaline protease activity (*P* < 0.05). Both the quadratic response and Tukey’s test showed reduced intestinal amylase and lipase activity when fish were fed with 230 g CBH kg^–1^ (*P* < 0.05) (Fig. 3).

## Discussion

Dietary CBH are essential as primary energy sources for farmed fish. However, excessive CBH inclusion generally impairs growth, whereas insufficient inclusion limits optimal performance (Sulaiman et al. 2020; Walker et al. 2020; Felix e Silva et al. 2022; Marchão et al. 2024). Therefore, providing adequate dietary energy within a balanced formulation ensures sufficient intake of essential amino acids (Batista et al. 2025), which are fundamental substrates for tissue growth.

In this study, the CBH content of the experimental isoproteic diets was progressively increased by reducing the inclusion of fish meal, corn bran, and wheat bran, while increasing toasted soybean meal and corn starch. Our findings indicate that dietary CBH levels of 260–290 g kg^–1^ optimize growth performance and whole-body nutrient deposition in red-bellied pacu. In contrast, diets containing 320 and 350 g kg^–1^ resulted in the poorest zootechnical outcomes. This pattern suggests that, like other omnivorous and herbivorous fish, the red-bellied pacu efficiently utilizes CBH when dietary levels do not exceed its metabolic capacity. Notably, the same CBH range (260–290 g kg^–1^) had a balanced amino acid composition and improved BPD, corroborating the observed growth enhancement and indicating that intermediate CBH inclusion supports efficient tissue synthesis by directing absorbed amino acids toward protein accretion rather than energy production (Ning et al. 2023). When dietary CBH adequately meets energy demands, protein catabolism is minimized, thereby enhancing growth potential.

Comparable results have been reported for omnivorous species such as Nile tilapia (optimal at ~ 300 g kg^–1^) (Boonanuntanasarn et al. 2018), pacu (~ 350 g kg^–1^) (Pereira et al. 2020), and hybrid lemon fin barb (*Barbonymus gonionotus* × *Hypsibarbus wetmorei*) (200–350 g kg^–1^) (Sulaiman et al. 2020), as well as for the herbivorous blunt snout bream (*Megalobrama amblycephala*) (200–350 g kg^–1^) (Li et al. 2013). Su et al. (2021) found that herbivorous grass carp (*Ctenopharyngodon idellus*) performed well at ~ 300 g CBH kg^–1^, whereas carnivorous Chinese longsnout catfish (*Leiocassis longirostris*) did not. In carnivorous rainbow trout (*Oncorhynchus mykiss*), it is recommended to keep CBH levels below 200 g kg^–1^ (Zhao et al. 2022). These interspecific differences reflect species-specific traits, feeding habits, CBH sources, and experimental conditions, reinforcing the ability of herbivorous and omnivorous fish to effectively digest and metabolize dietary CBH.

Conversely, excessive CBH intake may increase the HSI (Satiro et al. 2024), as observed in the present study. Elevated HSI values typically reflect liver enlargement associated with enhanced conversion of glucose into glycogen and lipids, which can disrupt normal metabolic processes (De Farias et al. 2021; Becker et al. 2024). We also observed increased VSI at dietary CBH levels of 260–320 g kg^–1^, but not at 350 g kg^–1^. Higher VSI generally reflects lipid accumulation in visceral tissues (Azaza et al. 2015), potentially reducing lipid deposition in the liver. However, the lower VSI at 350 g kg^–1^ does not necessarily indicate reduced hepatic lipid overload, as excessive CBH intake continues to promote hepatic lipid storage (Zheng et al. 2023), thereby impairing lipid metabolism and altering ATP and NADH homeostasis.

Patterns of whole-body total lipid content and BFD closely mirrored growth performance, with the highest values observed at intermediate CBH levels (260–290 g kg^–1^). This response may be attributed to increased availability of soluble CBH, which stimulates lipogenic enzymes such as glucose-6-phosphate dehydrogenase (G6PD) and 6PGDH, enhancing NADPH production, a key cofactor for fatty acid synthesis (Lin and Shiau 1995). Consequently, dietary CBH can be converted into lipids (Azaza et al. 2015; Satiro et al. 2024). Increasing CBH levels may therefore promote de novo fatty acid synthesis from non-lipid precursors, spare lipids from catabolism (Viegas et al. 2016; Sulaiman et al. 2020), and reduce fatty acid β-oxidation and lipoprotein clearance. The increased hepatic 6PGDH activity observed in fish fed high-CBH diets supports the association among elevated HSI, BFD, and plasma triglycerides, linking body-composition changes to metabolic adjustments.

The highest CBH diets also resulted in the lowest whole-body ash content and BAD. These diets contained reduced levels of fish meal, and since fish meal is recognized for its high mineral content and bioavailability, such outcomes were expected (Lall and Kaushik 2021). In contrastSandre et al. (2017b) reported that high CBH inclusion (460 g kg^–1^) in tambaqui (*Colossoma macropomum*) diets did not affect crude protein or ash content, but did improve nutritional efficiency and energy retention, highlighting species-specific responses in nutrient deposition.

Hematological profiles are often sensitive to dietary changes, providing valuable insights into growth, metabolism, and enzyme activity (Morante et al. 2021; Copatti et al. 2022). In the present trial, erythropoiesis appears to have occurred in the treatments that showed superior growth and whole-body deposition responses, as evidenced by increases in erythrocytes and MCV in fish fed 260 and 290 g CBH kg^–1^, respectively. Elevated MCV suggests erythropoietic stimulation, driving the production, differentiation, and maturation of red blood cells, which replace old cells (Seibel et al. 2021). In 260 g CBH kg^–1^ treatment, MCHC also decreased, indicating a change in the hemoglobin concentration within erythrocytes. However, the increase in the quantity of cells, associated with stable hemoglobin, hematocrit, and MCHC values, suggests a physiological adjustment to enhance the oxygen transport capacity, avoiding dehydration or impairment of cellular integrity in erythrocytes (Lemos et al. 2025), reinforcing the metabolic responses and, consequently, the growth of the fish in this treatment. Moreover, the absence of changes in hematocrit and MCH further indicates the absence of physiological stress, as reductions in these parameters are typically associated with malnutrition or infection (Seibel et al. 2021).

In contrast, higher CBH levels (320 and 350 g kg^–1^) altered hematological balance, with reductions in hemoglobin or erythrocyte counts and changes in MCHC, indicating shifts in red cell function and oxygen transport. These changes coincide with poorer growth performance and metabolic imbalance, reinforcing the link between hematological responses and overall physiological condition.

Although hematological parameters can reflect systemic responses, they have limitations and should be interpreted in conjunction with the other data provided in our work. For example, hematological indices are indirect markers; they don’t measure metabolic pathways, enzyme activity, or nutrient utilization (Wedemeyer 1996). Obviously, they also provide limited insight into organ-specific processes (e.g., liver, muscle, and intestine) that drive animal growth and metabolism (Hrubec and Smith 2010). So, improved fish growth performance does not necessarily correspond to detectable changes in these parameters, underscoring the importance of integrating hematological, biochemical, and enzymatic data to accurately assess fish physiological status.

Excessive dietary CBH intake can induce metabolic disturbances, including enhanced lipogenesis and elevated triglyceride levels (Satiro et al. 2024). In this study, high CBH diets increased plasma triglycerides and liver and muscle glycogen levels, likely due to upregulation of fatty acid biosynthesis and lipogenic pathways (Yang et al. 2020a). Despite these changes, glycemia remained stable, probably because the liver efficiently sequesters glucose for storage as glycogen or lipids and releases it in response to physiological demand (Sandre et al. 2017b). Thus, increased glucose intake does not necessarily result in hyperglycemia and may reflect an efficient regulation of glucose homeostasis (Walker et al. 2020; Taj et al. 2021). This reflects coordinated regulation of glucose uptake, storage, clearance, and production rather than a rise in glycemia (Dai et al. 2024). Plasma glucose after a CBH load often spikes transiently and then returns to baseline faster, especially after adaptation to high‑CBH diets or in more tolerant species (Polakof et al. 2008; Yang et al. 2020b). Adaptation to high‑CBH diets improves glucose tolerance by clearing plasma glucose more rapidly, via increased glycolytic enzyme activity and glycogen synthesis (Hemre et al. 2002; Dai et al. 2024).

Maintaining glucose homeostasis alongside increased glycogen reserves may reflect an adaptive metabolic shift toward glycolysis (Dai et al. 2024). However, when tissue glycogen accumulation exceeds tolerance thresholds, growth performance and feed efficiency may decline (Ning et al. 2023). This was confirmed in our study, as zootechnical performance deteriorated in parallel with increased muscle and liver glycogen, as well as plasma triglycerides, at higher CBH levels. In Nile tilapia, Azaza et al. (2015) similarly reported that CBH-rich diets stimulate insulin release, enhancing hepatic glycolytic enzyme activity (PK, PFK1) and glycogen storage to regulate blood glucose.

Beyond glycogen storage, glucose can also be converted into lipids (Felix e Silva et al. 2020; Yang et al. 2020b), consistent with the HSI results observed here. Elevated dietary CBH can activate insulin signaling, channeling excess glucose into glycogen and lipids (Taj et al. 2021). The elevated triglyceride levels indicate that increased CBH intake stimulated lipogenesis in red-bellied pacu (De Farias et al. 2021), while glycemia remained unchanged. Similar trends have been documented in tambaqui and Nile tilapia, where excess glucose derived from CBH was converted into fatty acids and triglycerides, which were subsequently stored in the liver or released into circulation (Sandre et al. 2017b; Felix e Silva et al. 2020; Satiro et al. 2024). Excessive lipid accumulation can impair fish physiology by stimulating pro-inflammatory cytokines and inducing insulin resistance, thereby hindering growth and development (Zhang et al. 2024).

In fish, amino acids originating from dietary or tissue protein breakdown undergo deamination, with the resulting intermediates subsequently being oxidized, thereby influencing metabolic regulation (Arenas et al. 2021; De Farias et al. 2021; Marchão et al. 2024). In the present study, hepatic AST and GDH activities decreased in fish fed diets rich in CBH. Diets low in CBH and high in protein or limited in energy typically increase aminotransferase activity, particularly AST, reflecting enhanced protein mobilization (Metón et al. 1999). Under such conditions, GDH activity, another key enzyme in amino acid catabolism, also tends to increase (Thongchaitriwat et al. 2024). Thus, the reduced AST and GDH activities observed here suggest that dietary protein was preferentially allocated to muscle accretion rather than used as a gluconeogenic substrate (Boonanuntanasarn et al. 2018). Efficient energy utilization may therefore spare amino acids required for maintaining physiological stability and acid–base balance (Copatti et al. 2019).

As reported by Carli et al. (2024), reliance on protein catabolism to meet energy demands increases the activity of gluconeogenic enzymes, such as FBPase1, as well as AST and GDH. Gluconeogenesis, which synthesizes glucose from non-CBH precursors, is heavily regulated by FBPase1 in fish (Melo et al. 2016) and generally predominates when dietary CBH availability is low, supplementing glucose and glycogen production from protein or lipid sources. Consequently, replacing dietary protein with CBH may lead to lower AST activity, as observed in our work and similarly reported in the European eel (Suárez et al. 2002), gilthead seabream (Caseras et al. 2002), rainbow trout (Kirchner et al. 2005), and tambaqui (Carli et al. 2024). In contrast, serum AST activity increased in largemouth bass (*Micropterus salmoides*)—a carnivorous species with poor CBH utilization—when fed high-CBH diets (Zou et al. 2025). Enhanced glycolysis coupled with suppressed gluconeogenesis facilitates CBH utilization (Su et al. 2021), whereas gluconeogenesis predominates when dietary CBH is poorly metabolized (Melo et al. 2016). These findings are consistent with our glycemia data, which show that downregulation of gluconeogenesis allows excess dietary CBH to be stored rather than persisting as hyperglycemia (Hemre et al. 2002; Taj et al. 2021; Dai et al. 2024).

Non-carnivorous fish exhibit physiological adaptations that enhance CBH utilization, including improved glucose regulation and increased intestinal enzyme activity (Su et al. 2020). In this study, intestinal amylase and lipase activities were higher in fish fed intermediate- and high-CBH diets than in those fed the control diet. Amylase activity is considered a key indicator of CBH digestion efficiency (Su et al. 2020), and elevated amylase activity linked to glut2 expression has been reported in grass carp fed high-CBH diets (Su et al. 2021). The increased amylase activity observed here suggests enhanced CBH digestion efficiency (De Souza et al. 2021b). Although lipase activity is primarily associated with lipid hydrolysis and may not be directly related to CBH levels (Copatti et al. 2022; Marchão et al. 2025), CBH can influence gut microbiota composition, stimulating the production of diverse enzymes or enhancing host digestive enzyme activity (Zhang et al. 2024), which may partially explain the elevated lipase and amylase activities observed with increased CBH intake.

Conversely, alkaline protease activity declined as dietary CBH levels increased, suggesting that more amino acids were available for growth rather than catabolism (Morante et al. 2021; Marchão et al. 2025). This reduction likely results from CBH meeting most of the energetic requirements, thereby diminishing protein degradation and associated protease activity (Wang et al. 2022). Additionally, intestinal microbiota may modulate CBH metabolism by regulating the expression of genes involved in glycolysis and gluconeogenesis (Zhang et al. 2024). Consistently, we observed higher hepatic activities of glycolytic (PFK1 and PK) and tricarboxylic acid cycle (ICDH) enzymes in red-bellied pacu fed diets with elevated CBH content, indicating a preference for CBH as the primary energy source.

Comparable increases in glycolytic and lipogenic enzyme activities have been reported in Nile tilapia (Boonanuntanasarn et al. 2018; Thongchaitriwat et al. 2024), yellow catfish (Yang et al. 2020a), pacu (Pereira et al. 2020), and tambaqui (Carli et al. 2024) under high-CBH feeding regimes. While certain carnivorous fish exhibit poor CBH utilization, many omnivorous and herbivorous species naturally consume CBH-rich diets and can adapt metabolically to such feeding strategies by downregulating gluconeogenic enzymes and upregulating glycolytic and lipogenic pathways (Li et al. 2018; Kumkhong et al. 2021; Carli et al. 2024). These metabolic adjustments were evident in red-bellied pacu, confirming their capacity to adapt efficiently to CBH-rich diets, particularly within the 260–290 g CBH kg^–1^ range. Within this range, energy supply is balanced, amino acids are spared for growth, and metabolic processes remain regulated (Jiang et al. 2018; Naiel et al. 2022). Nevertheless, CBH inclusion above 320 g kg^–1^ disrupts this balance, leading to metabolic overload, altered hematological profiles, impaired lipid metabolism, and reduced growth performance.

## Conclusions

The present study demonstrates that dietary CBH levels between 260 and 290 g kg^–1^ promote optimal growth performance, whole-body nutrient deposition, and digestive enzyme activity in red-bellied pacu, as indicated by derived quadratic equations. Within this range, regression analysis and Tukey’s test showed that CBH efficiently meets energy requirements, supporting physiological stability as reflected by hematological parameters and plasma glucose homeostasis. In addition, adequate CBH inclusion enhances glucose utilization by stimulating glycolysis and lipogenesis while suppressing gluconeogenesis, thereby sparing dietary protein and minimizing amino acid catabolism, as evidenced by reduced transamination and deamination activities. In contrast, diets containing more than 320 g CBH kg^–1^ are not recommended, as they are associated with impaired growth, increased BFD, and higher FCR, which can potentially compromise fish metabolism and feed efficiency.

## Supplementary Information

Below is the link to the electronic supplementary material.

Supplementary Material 1

## Data Availability

The data supporting this study’s findings are available from the corresponding author upon reasonable request.

## Abbreviations

PFK1: 6-Phosphofructo–1-kinase
6PGDH: 6-Phosphogluconate dehydrogenase
ANOVA: Analysis of variance
AST: Aspartate aminotransferase
ATP: Adenosine triphosphate
BAD: Body ash deposition
BFD: Body fat deposition
BPD: Body protein deposition
CBH: Carbohydrate
CY: Carcass yield
FCR: Feed conversion ratio
FBPase1: Fructose−1,6‐bisphosphatase 1
GDH: Glutamate dehydrogenase
HSI: Hepatosomatic index
ICDH: Isocitrate dehydrogenase
MCHC: Mean corpuscular hemoglobin concentration
MCV: Mean corpuscular volume
MCH: Mean corpuscular hemoglobin
NADH: Nicotinamide adenine dinucleotide
NADPH: Nicotinamide adenine dinucleotide phosphate
PK: Pyruvate kinase
SGR: Specific growth rate
VSI: Viscerosomatic index
